# Plasma and synovial fluid concentrations of linezolid in patients with knee osteoarthritis infected with *Staphylococcus aureus*

**DOI:** 10.1186/s40780-022-00248-9

**Published:** 2022-07-01

**Authors:** Daisuke Negishi, Okimichi Mitsumatsu, Takahiro Matsumura, Hiromi Mitsumatsu, Miaki Makiguchi, Makiko Shimizu, Hiroshi Yamazaki

**Affiliations:** 1 Showa Pharmaceutical University, 3-3165 Higashi-tamagawa Gakuen, Machida, Tokyo, 194-8543 Japan; 2 Kamakura Hospital, 1-8 Hase 3-chome, Kamakura, Kanagawa 248-0016 Japan

**Keywords:** Drug monitoring data, Knee osteoarthritis, Synovial fluid penetration

## Abstract

**Background:**

Linezolid is a new oxazolidinone antibiotic used for infections caused by methicillin-resistant *Staphylococcus* and other species.

**Case presentation:**

Two cases of knee osteoarthritis with acute infection were successfully treated using linezolid. The plasma and synovial fluid concentrations of linezolid in two patients [women aged 69 and 73 years (cases 1 and 2)] with knee osteoarthritis infected with *Staphylococcus aureus* were measured after they were administered 600 mg twice daily by intravenous infusion. The plasma linezolid concentrations during knee surgery in case 1 at day 5 and in case 2 at day 2 were 19.6 and 15.6 μg/mL, respectively. The synovial fluid concentrations of linezolid in samples taken during surgery in case 1 and case 2 were 14.9 and 17.0 μg/mL, respectively; these values corresponded to ratios of synovial fluid/plasma of 76 and 109%. Possible metabolite 2-hydroxylated linezolid potentially mediated by cytochrome P450 2 J2 was not detected in the plasma or synovial fluid samples under the current clinical setting after multiple doses.

**Conclusions:**

These results implied nearly equivalent concentrations of linezolid in plasma and synovial fluid of clinical patients with knee osteoarthritis acutely infected with *Staphylococcus aureus*.

## Background

Linezolid is a new oxazolidinone antibiotic used for infections caused by methicillin-resistant *Staphylococcus* and other species [1, 2]. Linezolid is a promising antibiotic drug, but its use is limited by adverse effects with prolonged administration of 600 mg twice daily [3–5]. The pharmacokinetics of linezolid in healthy subjects [6] and in obese patients with cellulitis [7] have been reported. Linezolid is reportedly hydroxylated by as-yet unidentified cytochrome P450s [8], but recently a role for P450 2 J2 in linezolid 2-hydroxylation was indicated [9].

The case of a patient with chronic infection of an orthopedic implant treated with linezolid has been reported [10]. In the current study, two cases of acute infection of knee osteoarthritis were successfully treated using linezolid. From a clinical perspective, the monitoring of synovial fluid concentrations of linezolid is of interest. Synovial fluid concentrations of linezolid assessed during knee surgery in these two patients were determined and the ratios of synovial fluid/plasma were calculated.

## Case presentation

Two cases of acute infection of knee osteoarthritis were successfully treated using linezolid between August 2018 and February 2019. The plasma and synovial fluid concentrations of linezolid in two patients (women aged 68 and 73 years, Table 1) treated in Kamakura Hospital for knee osteoarthritis infected with *Staphylococcus aureus* were determined after administration of 600 mg twice daily by intravenous infusion (at 6 am and 6 pm) for 11 days in case 1 and 19 days in case 2. Clinical laboratory results before linezolid treatment for case 1 were serum creatinine (0.61 mg/dL), aspartate aminotransferase (32 IU/L), alanine aminotransferase (37 IU/L), serum albumin (4.1 g/dL), and platelet (36.5 × 10^4^/μL); and those for case 2 were serum creatinine (0.40 mg/dL), aspartate aminotransferase (22 IU/L), alanine aminotransferase (31 IU/L), serum albumin (3.0 g/dL), and platelet (30.0 × 10^4^/μL). Co-administrated drugs during linezolid treatment for both cases were loxoprofen (180 mg per day from day1) and lansoprazole (15 mg from day1), with additional clindamycin (900 mg from day 2) for case 2. The patients were discharged 25 days (case 1) and 41 days (case 2) after admission. The patients gave written informed consent to take part in this study and for its publication. The Ethics Committee of Kamakura Hospital approved this study. The synovial fluid samples collected from the two patients during surgery along with plasma samples were pharmacokinetically analyzed. Samples (50 μL) of both plasma and synovial fluid taken from the two above-described patients were treated with an equal volume of acetonitrile, and the aqueous supernatant was centrifuged at 15,000×*g* for 10 min at 4 °C.

**Table 1:** Plasma and synovial fluid concentrations of linezolid determined in two patients during knee surgery

Case (body weight)		Sampling	Linezolid concentration (μg/mL)		Ratio of synovial fluid/plasma
			Plasma	Synovial fluid	
1	69-year-old woman (50 kg)	Day 5	19.6 ± 0.9	14.9 ± 0.4	76%
2	73-year-old woman (50 kg)	Day 2	15.6 ± 0.2	17.0 ± 0.5	109%

Data are means ± standard deviations from triplicate determinations

**Fig. 1:**
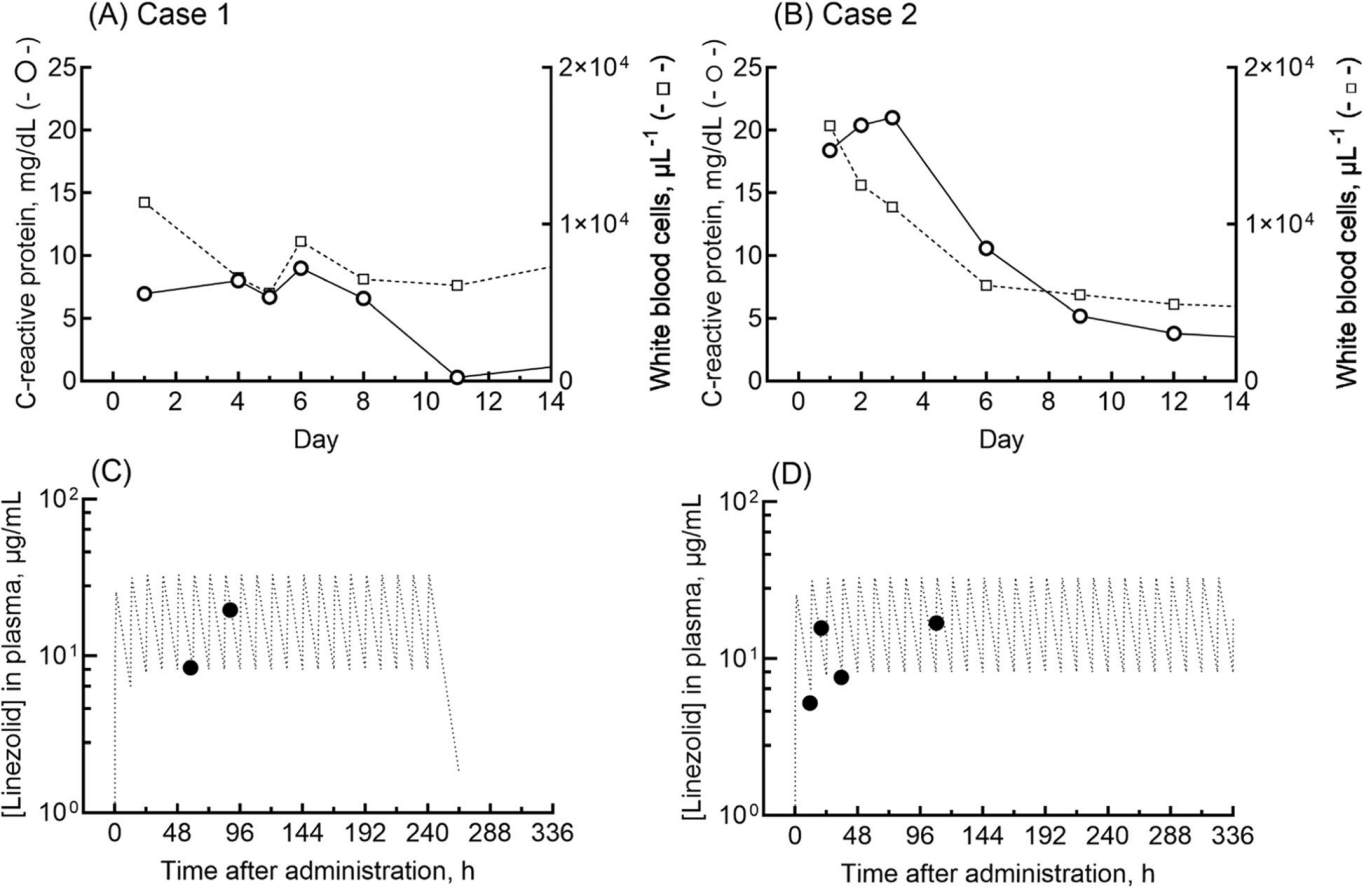
Clinical laboratory results and measured plasma concentrations of linezolid in two patients administered 600 mg twice daily by intravenous infusion. Levels of C-reactive protein (circles, mg/dL) and white blood cell counts (squares, μL^− 1^) in two patients are indicated: (**A**) a 69-year-old woman, case 1, and (**B**) a 73-year-old woman, case 2. Measured plasma concentrations of linezolid (closed circles) in case 1 (**C**) and case 2 (**D**) after administrations are shown. Also shown are the plasma concentrations of linezolid (dashed lines) after virtual daily doses generated using a previously reported one-compartment model [11]

The linezolid levels in plasma and synovial fluid samples from the two patients (Fig. 1) were quantified using liquid chromatography

Under the current clinical setting, it was focused to find any hydroxylated linezolid metabolite(s) in the plasma or synovial fluid samples. To investigate the possible P450 2 J2-dependent 2-hydroxylation of linezolid [9], an incubation mixture was prepared consisting of 100 pmol/mL recombinant P450 2 J2 protein (Corning, Woburn, MA, USA), 0.40 mM linezolid, an NADPH-generating system (0.50 mM NADP^+^, 5.0 mM glucose 6-phosphate, and 0.50 units/mL glucose 6-phosphate dehydrogenase), and 100 mM potassium phosphate buffer (pH 7.4) in a total volume of 0.20 mL. The reaction was carried out at 37 °C for 60 min and was terminated by adding 0.60 mL of acetonitrile. The reaction mixture was then centrifuged at 15,000×*g* for 10 min.

Plasma and synovial fluid concentrations of linezolid and that of the in vitro product of linezolid and P450 2 J2 as a possible metabolite were determined using a reverse-phase high-performance liquid chromatography system (Shimadzu, Kyoto, Japan) with a Mightysil RP-18GP Aqua column (5 μm, 150 × 4.6 mm, Kanto Chemical, Tokyo, Japan) equilibrated in a mobile phase comprising 10% CH_3_CN in 0.1% aqueous formic acid at a flow rate of 1.5 mL/min and a column temperature of 45 °C with ultraviolet monitoring at 254 nm. This mobile phase composition was held for 1 min followed by a linear gradient to 50% CH_3_CN at 8 min, a second linear gradient to 62% CH_3_CN at 10 min, a 5 min wash at 90% CH_3_CN, followed by re-equilibration at the initial conditions for another 4 min. Samples (10 μL) were infused using an autosampler. The retention times of linezolid and its possible 2-hydroxylated metabolite [9] were 7.4 and 5.9 min respectively. Data are given as means and standard deviations from triplicate determinations.

The clinical laboratory results for these two patients over a 2-week period are shown in Fig. 1A and B. The patient in case 2 was found by outsourced clinical laboratory services to be suffering from methicillin-resistant *Staphylococcus aureus* infection*,* but in case 1, the infectious agent was methicillin-sensitive *Staphylococcus aureus.* For cases 1 and 2, the measured plasma concentrations of linezolid and the pharmacokinetic-modeled concentration profiles of linezolid (obtained using a previously reported one-compartment model with the following parameters of absorption constant, 0.126 h^− 1^; volume of distribution, 22 L; and half-life, 5.5 h) are shown in Fig. 1C and D**,** respectively [11]. The plasma linezolid concentrations in samples taken during knee surgery in case 1 at day 5 and in case 2 at day 2 were 19.6 and 15.6 μg/mL, respectively.

**Fig. 2:**
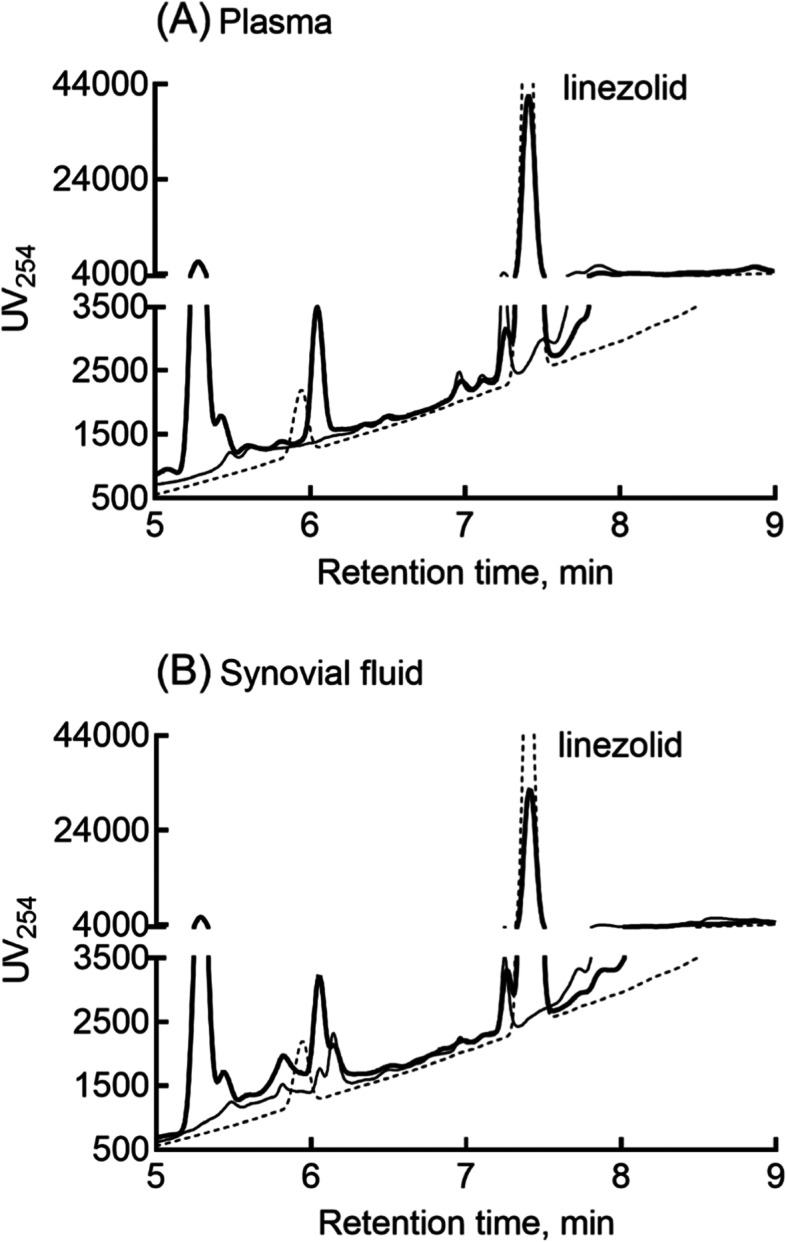
Representative chromatograms for linezolid in plasma and linezolid in synovial fluid. Plasma (**A**) and synovial fluid (**B**) samples from case 1 before (thin lines) and after (thick lines) treatment with linezolid twice a day were investigated. Possible linezolid 2-hydoxylated metabolite(s) mediated by recombinant human P450 2 J2 in an in vitro system (dashed lines) was used for chromatographic comparison. The retention times of linezolid and its possible 2-hydroxylated metabolite [9] were 7.4 and 5.9 min respectively

Figure 2 shows representative chromograms for linezolid in plasma and synovial fluid. There were additional polar peaks to that of substrate linezolid alone in both plasma and synovial fluid samples from the patient after infusion of linezolid. However, the presence of 2-hydroxylated linezolid potentially mediated by cytochrome P450 2 J2 was not detected in these plasma or synovial fluid samples in the current clinical setting (Fig. 2). Synovial fluid concentrations of linezolid taken during knee surgery in case 1 and case 2 were 14.9 and 17.0 μg/mL, respectively, which corresponded to ratios of synovial fluid/plasma of 76 and 109% (Table 1).

## Discussion and conclusion

Linezolid is reportedly not highly protein-bound (30%) and has a moderate volume of distribution similar to the total water content of the body [12]. Relatively good penetration of linezolid into inflammatory blister fluid has been demonstrated in healthy volunteers [13, 14]. It also has been reported that linezolid concentrations in soft tissue specimens ranged from 18 to 78% (mean, 51%) of their simultaneous serum levels in diabetic patients with foot infections [11]. Plasma-to-synovial-fluid ratios of 0.76 ± 0.34 for linezolid have been reported in terms of the knee gap after a single dose of 600 mg of linezolid [15]. Although the package insert information of linezolid suggests the presence of two minor morpholine ring-opening metabolites in human plasma or urine (in-house document), recently reported 2-hydroxylated linezolid (mediated by cytochrome P450 2 J2 [9]) was not considered so far, to our knowledge, in the fluid samples in the clinical setting. In the current study, similar plasma and synovial fluid concentrations of parent compound linezolid in two patients with knee osteoarthritis infected with *Staphylococcus aureus* were demonstrated after patients were administered multiple doses of 600 mg by twice daily intravenous infusion. The present results suggested almost equivalent concentrations of linezolid in plasma and synovial fluid of clinical patients with knee osteoarthritis infected with *Staphylococcus aureus* after multiple doses. A population-based pharmacokinetic model of linezolid in hospitalized patients with chronic arthritis has been proposed [16]. Simple systematic therapeutic drug monitoring for linezolid [17], which may reflect the concentrations of linezolid in synovial fluid after multiple doses, would have a beneficial clinical impact on the treatment of synovial fluid infections in patients with knee osteoarthritis.

## Data Availability

All data generated or analyzed during this study are included in this published article and are also available from the corresponding author on reasonable request.

## Abbreviation

P450: cytochrome P450
